# Racial/Ethnic Disparities on the Risk of Second Malignant Neoplasm Among Hodgkin Lymphoma Survivors

**DOI:** 10.3389/fonc.2021.790891

**Published:** 2022-01-24

**Authors:** Han Xiao, Jianghua He, Simin Liang, Duo Cai, Qiao Zhou, Lanxiang Liu, Xinyu Yan, Jianxiang Chi, Qing Xiao, Li Wang

**Affiliations:** ^1^ Department of Hematology, The First Affiliated Hospital of Chongqing Medical University, Chongqing Medical University, Chongqing, China; ^2^ Department of Biostatistics and Data Science, University of Kansas Medical Center, Kansas City, KS, United States; ^3^ The Center for the Study of Hematological Malignancies, Karaiskakio Foundation, Nicosia, Cyprus

**Keywords:** Hodgkin lymphoma, second malignant neoplasm, SEER database, racial/ethnic disparities, cancer surveillance

## Abstract

**Background:**

Hodgkin lymphoma survivors are at risk for second malignant neoplasm (SMN). How race/ethnicity affects the risk remains unclear.

**Methods:**

This retrospective cohort study included 22,415 patients diagnosed with primary Hodgkin lymphoma from January 1992 to December 2015 in 13 Surveillance, Epidemiology, and End Results-based registries and divided patients into four groups: non-Hispanic whites, non-Hispanic blacks, Hispanics, and Asian/others. Taking non-Hispanic whites as a reference, both the proportional subdistribution hazard (PSH) and the cause-specific hazard (CSH) methods were used to calculate the SMN hazard ratio for other racial/ethnic groups with and without considering the competing mortality risk.

**Results:**

1,778 patients developed SMN with a median follow-up of 11.63 years. In the adjusted PSH model, Hispanic, Asian/others, and non-Hispanic black patients had 26% (PSH, 0.74; 95% CI, 0.63–0.87), 20% (PSH, 0.80; 95% CI, 0.64–1.01), and 12% (PSH, 0.88; 95% CI, 0.75–1.03) decreased overall SMN hazard, respectively. Moreover, the PSH method revealed the racial/ethnic difference in the SMN risk in the skin, the respiratory system, and the endocrine system. These hazards were slightly higher and different with the use of the CSH approach. In addition to the aforementioned overall SMN and subtypes, adjusted CSH analysis also revealed the racial/ethnic disparities in the risk of subsequent female breast cancer, digestive cancer, and non-Hodgkin lymphoma.

**Conclusions:**

The subtype and SMN risk among Hodgkin lymphoma survivors varied by race/ethnicity. The use of CSH and PSH provides a dynamic view of racial/ethnic effects on SMN risk in Hodgkin lymphoma survivors.

## Introduction

Hodgkin lymphoma is a group of lymphoid neoplasms in which cancerous Reed–Sternberg cells are mixed with heterogeneous inflammatory cells, accounting for approximately 10% of all lymphomas, 0.6% of all cancers, and 0.2% of all cancer mortalities (1–3). Over the previous century, advances in treatment have drastically improved the survival of Hodgkin lymphoma patients wherein most patients will be cured (4, 5). However, growing long-term Hodgkin lymphoma survivors are at risk for late complications (e.g., second malignancies). Studies have demonstrated that Hodgkin lymphoma survivors have a higher risk of developing solid tumors and hematologic malignancies than the general population (6, 7). These second malignant neoplasms (SMNs) significantly impact the long-term survival of Hodgkin lymphoma patients (8, 9).

The risk of developing an SMN in Hodgkin lymphoma patients depends on factors related to the patient and the treatment, including age at treatment, family cancer history, smoking history, and the effect of treatment given (10–17). However, considerable racial/ethnic differences exist in these risk factors for SMN among Hodgkin lymphoma patients. The mean age of Hodgkin lymphoma diagnosis among whites was significantly older than all other races. The peak incidence of Hodgkin lymphoma was in young adulthood among non-Hispanics but was in the elderly among Hispanics (18). Moreover, whites were more likely to have family cancer information documented than non-whites (19, 20). The smoking prevalence also varied by race/ethnicity. Individuals of white and black descent have been reported to have a higher smoking prevalence than individuals of Asian and Hispanic/Latino descent (21). The study results about the association between treatment selection and race/ethnicity in Hodgkin lymphoma patients are not consistent. Rodday et al. showed that race/ethnicity was not associated with first-line treatment received using the SEER-Medicare database (22). However, Olszewski et al. reported that black and Hispanic patients received radiotherapy less frequently than white patients (23). Given this potential difference in clinical factors, SMN risk could also differ by race/ethnicity, which has important clinical implications on the long-term follow-up of Hodgkin lymphoma survivors.

The cause-specific hazard (CSH) is a classic method to ascertain the disease etiology and yields valid associations, which can be an ideal way to evaluate the direct association between race/ethnicity and SMN among Hodgkin lymphoma survivors without considering the effects of competing events. However, in the real world, mortality due to other causes can prevent from observing the SMN occurrence. A previous study showed that non-Hispanic black and Hispanic children had worse overall survival than non-Hispanic white patients (24). The difference in mortality may influence the actual racial-ethnic-specific SMN rate among Hodgkin lymphoma survivors. The proportional subdistribution hazard (PSH) is a more appropriate way to reveal how the probability of developing SMN differed by race/ethnicity in the actual situation (25, 26). With data from the National Cancer Institute Surveillance, Epidemiology, and End Results (SEER) Program, the present study would examine the effects of race/ethnicity on SMN risk in Hodgkin lymphoma survivors by PSH and CSH methods, with and without considering competing risks of mortality. As suggested by Latouche et al., the hazards of competing events (mortalities due to other causes) were also presented for complete understanding (27, 28).

## Materials and Methods

### Data Source and Cohort Selection

**Figure 1:**
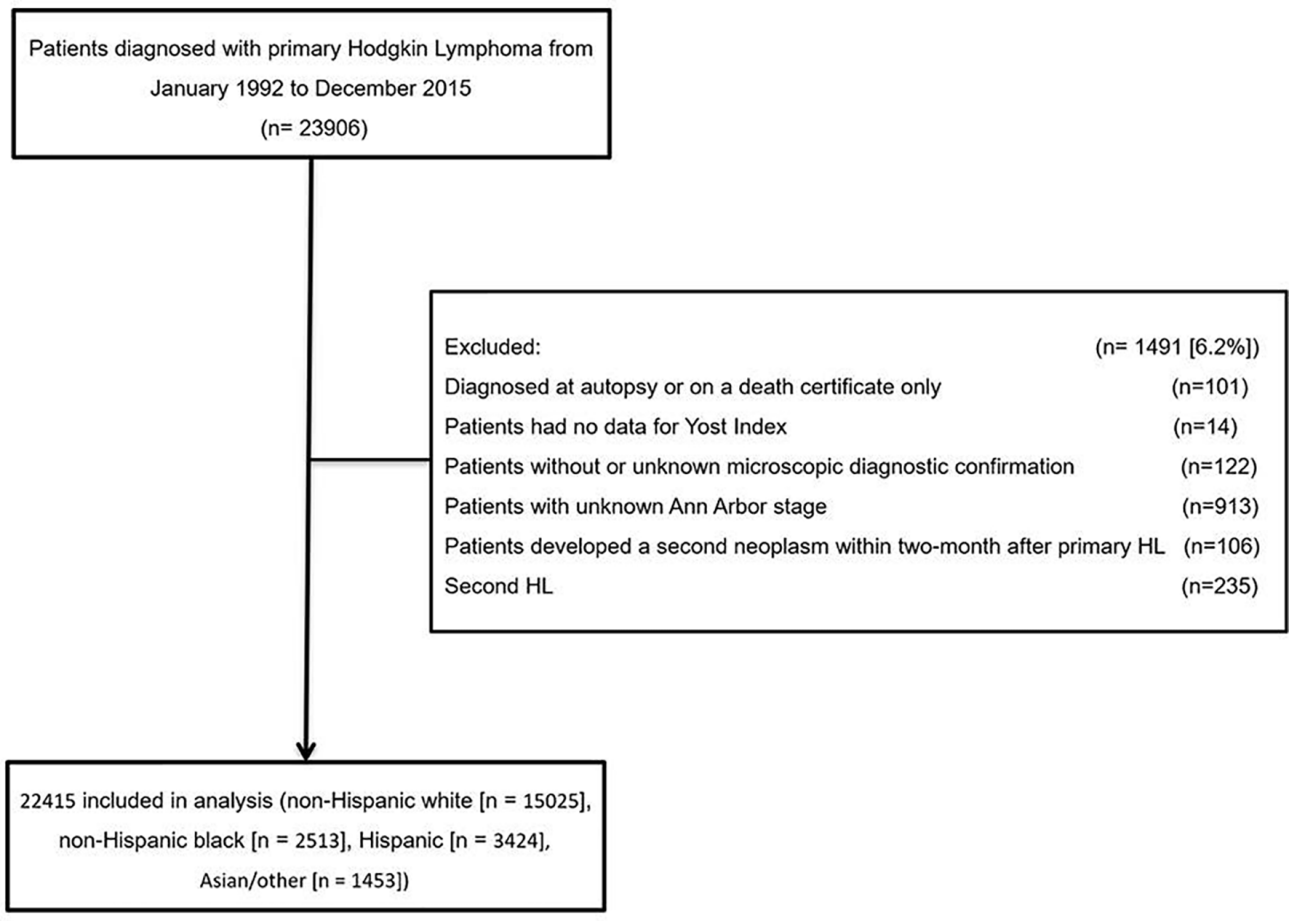
Cohort selection. *HL*, Hodgkin lymphoma.

A retrospective cohort study using data from 13 SEER cancer registries, Nov 2018 Submission, which covers approximately 13.4% of the US population, was conducted. This analysis included patients diagnosed with primary Hodgkin lymphoma from January 1992 to December 2015 (*n* = 23,906). Eligible patients were identified using the International Classification of Diseases for Oncology, third edition (ICD-O-3) morphology codes (Hodgkin lymphoma, 9,650–9,669). Patients diagnosed at autopsy or on a death certificate only (*n* = 101), had no data for Yost index (*n* = 14), without or unknown microscopic diagnostic confirmation (*n* = 122), and with unknown Ann Arbor stage were all excluded (*n* = 913). Moreover, patients who developed a second neoplasm within 2 months of the primary lesion were also excluded for the difficulty to identify which cancer was the first index cancer (*n* = 106). Patients who developed subsequent Hodgkin lymphoma were excluded for the difficulty to distinguish between second primary tumors and a recurrence (*n* = 235). The final sample size is 22,415 (**Figure 1**). The present study did not need ethics committee approval as the data are de-identified and publicly available.

The criteria for defining SMNs differ between studies (6, 7, 13, 14, 29). The definitions provided by the SEER project and the International Association of Cancer Registries and the International Agency for Research on Cancer (IACR/IARC) are widely used (30). Rules by SEER suggest the registration of synchronous tumors diagnosed in less than 2 months, which is used in the present study (31). However, IACR/IARC recommends using 6 months to distinguish between synchronous and metachronous multiple primaries (32). To test the overall impact of applying different definitions of SMN in the overall results of the present study, a sensitivity analysis was conducted with the rules developed by IACR/IARC.

Race or ethnicity is divided into non-Hispanic whites, non-Hispanic blacks, Hispanic, and Asian/others (which included non-Hispanic Asians, non-Hispanic Native American or Alaskans, non-Hispanic Native Hawaiians or other Pacific Islanders, and people of unknown racial or ethnic origin). Socioeconomic status was estimated using the Yost index, developed by Kathleen Yost, to evaluate the potential impact of socioeconomic gradients on cancer burden (33). Thus, a higher Yost score represents a higher socioeconomic status level.

### Statistical Analysis

Patients were observed from the time of diagnosis with primary Hodgkin lymphoma until diagnosis with SMN, mortality, last follow-up, or end of the study, whichever occurred first. The median follow-up time was calculated by the reverse Kaplan–Meier estimator (34). The cumulative SMN incidence was depicted using the PSH and CSH methods, respectively (35, 36). Both the CSH and PSH regression models were used to assess the effects of race/ethnicity on SMN risk on Hodgkin lymphoma survivors. Models were performed unadjusted (model 1); adjusted for age, gender, year of Hodgkin lymphoma diagnosis, Ann Arbor stage, and histology subtype (model 2); adjusted for age, gender, year of Hodgkin lymphoma diagnosis, Ann Arbor stage, histology subtype, and additionally Yost index (model 3); and adjusted for age, gender, year of Hodgkin lymphoma diagnosis, Ann Arbor stage, histology subtype, Yost index, and additionally treatment information (model 4). Baseline age (≤35 years, >35 years), sex (female, male), year of Hodgkin lymphoma diagnosis (1992–2003, 2004–2015), Ann Arbor Stage (I and II, III and IV), histology subtype of Hodgkin lymphoma (classic, non-classic), Yost Index (low, high), chemotherapy (yes, no/unknown), and radiotherapy (yes, no/unknown) were modeled categorically. The potential for multicollinearity was assessed using the variance inflation factor, with values between 1 and 5 considered acceptable (37, 38).

Among patients with SMN, the Cochran Armitage trend tests for trends was performed to evaluate trends in solid tumor proportions over time (39). SMNs in the present report are categorized on the basis of SEER site recode ICD-O-3/WHO 2008 definitions, which were recategorized into 12 different categories, as described in **Supplemental Table S1**. Both PSH and CSH methods were used to assess the racial/ethnic effects on the risk of categorized SMN subtypes. All reported *p* values were two-sided, and *p* values of <0.05 were considered statistically significant. All the analyses were conducted using R software version 4.03.

## Results

### Study Population and Cohort Selection

**Table 1** lists the baseline characteristics of included Hodgkin lymphoma patients. Among 22,415 patients, 67.03% of the cohort were non-Hispanic whites (*n* = 15,025), 11.21% were non-Hispanic blacks (*n* = 2,513), 15.28% were Hispanics (*n* = 3,424), and 6.48% were Asian/others (non-Hispanic Asians [*n* = 1,121], non-Hispanic Native American or Alaskans [*n* = 84], non-Hispanic Native Hawaiians or other Pacific Islanders [*n* = 119], and people of unknown racial or ethnic origin [n = 129]). The median age at primary Hodgkin lymphoma diagnosis was 35 years. Non-Hispanic white patients tended to be older than any other race/ethnicity (*p* < 0.001). Hispanic and non-Hispanic black patients had a lower Yost index than non-Hispanic white and Asian/other patients (*p* < 0.001). Moreover, the proportion of receiving radiotherapy and chemotherapy was the lowest in non-Hispanic black patients (*p* < 0.001). The proportion of nodular lymphocyte-predominant Hodgkin lymphoma subtype was the greatest for non-Hispanic black patients, followed by Asian/others, non-Hispanic whites, and then Hispanics (*p* < 0.001).

**Table 1:** Characteristics of patients with Hodgkin lymphoma in the SEER database by race and ethnicity (n = 22,415), diagnosed 1992–2015.

	Non-Hispanic White	Non-Hispanic Black	Hispanic	Asian/other	*p*
n	15,025	2,513	3,424	1,453	
Age, year (median [IQR])	36 [25, 52]	34 [25, 47]	32 [22, 50]	31 [23, 48]	<0.001
Gender = male, no. (%)	8,234 (54.8)	1,354 (53.9)	1,952 (57.0)	787 (54.2)	0.06
Diagnosis year = 2004–2015, no. (%)	7,386 (49.2)	1,425 (56.7)	1,998 (58.4)	925 (63.7)	<0.001
Yost index (median [IQR])	11,477 [11,045–11,604]	11,259 [10,936–11,556]	11,050 [10,964–11,551]	11,567 [11,050– 11,665]	<0.001
Histology, no. (%)					<0.001
cHL, NOS	2,377 (15.8)	529 (21.1)	684 (20.0)	276 (19.0)	
LD	149 (1.0)	25 (1.0)	68 (2.0)	20 (1.4)	
MC	1,885 (12.5)	318 (12.7)	584 (17.1)	167 (11.5)	
LR	432 (2.9)	88 (3.5)	98 (2.9)	50 (3.4)	
NS	9,515 (63.3)	1,308 (52.0)	1,874 (54.7)	874 (60.2)	
NLPHL	667 (4.4)	245 (9.7)	116 (3.4)	66 (4.5)	
Ann Arbor stage, no. (%)					<0.001
Stage I	3,114 (20.7)	515 (20.5)	581 (17.0)	242 (16.7)	
Stage II	6,457 (43.0)	868 (34.5)	1,279 (37.4)	661 (45.5)	
Stage III	3,027 (20.1)	559 (22.2)	743 (21.7)	269 (18.5)	
Stage IV	2,427(16.2)	571 (22.7)	821 (24.0)	281 (19.3)	
Radiotherapy, no. (%)					<0.001
Yes	6,172 (41.1)	749 (29.8)	1,043 (30.5)	616 (42.4)	
No/unknown	8,853(58.9)	1,764 (70.2)	2,381 (69.5)	837(57.6)	
Chemotherapy, no. (%)	15,025	2,513	3,424	1,453	<0.001
Yes	12,022 (80.0)	1,975 (78.6)	2,856 (83.4)	1,194 (82.2)	
No/unknown	3,003 (20.0)	538 (21.4)	568 (16.6)	259(17.8)	
Median person-years at risk [IQR]	12.55 [6.96, 18.71]	10.92 [5.80,17.05]	9.30 [4.38, 15.71]	9.38 [4.80;14.96]	<0.001

Categorical variables were compared using Pearson chi-square tests; continuous variables were compared using Kruskal–Wallis H tests.

cHL, classic Hodgkin lymphoma; NOS, not otherwise specified; LD, lymphocyte depleted; MC, mixed cellularity; LR, lymphocyte rich; NS, nodular sclerosing; NLPHL, nodular lymphocyte-predominant Hodgkin lymphoma; IQR, interquartile range.

### SMNs and Mortality in Hodgkin Lymphoma Survivors

The numbers of Hodgkin lymphoma patients experiencing SMN events and mortality without experiencing SMN are shown in **Supplemental Table 2**. With a median follow-up of 11.63 years, 1,778 and 4,774 patients developed second cancer and expired without experiencing an SMN, respectively. The 10-year cumulative incidence of SMNs was the highest for non-Hispanic white patients (6.58%; 95% CI, 5.91–7.25), followed by non-Hispanic black patients (5.35%; 95% CI, 4.35–6.35), Asian/others (5.12%; 95% CI, 3.78–6.45), and Hispanics (4.80%; 95% CI, 3.93–5.67). Moreover, the 10-year cumulative incidences of mortality without SMN were 19.17% (95% CI, 18.50–19.84), 20.44% (95% CI, 18.14–22.75), 24.53% (95% CI, 22.89–26.16), and 25.79% (95% CI, 23.91–27.67) in non-Hispanic whites, Asian/others, Hispanics, and non-Hispanic blacks, respectively. The gap between cumulative overall SMN incidence and mortality was the smallest among the non-Hispanic whites than any other racial/ethnic subgroups (**Supplemental Figure 1**).

**Figure 2:**
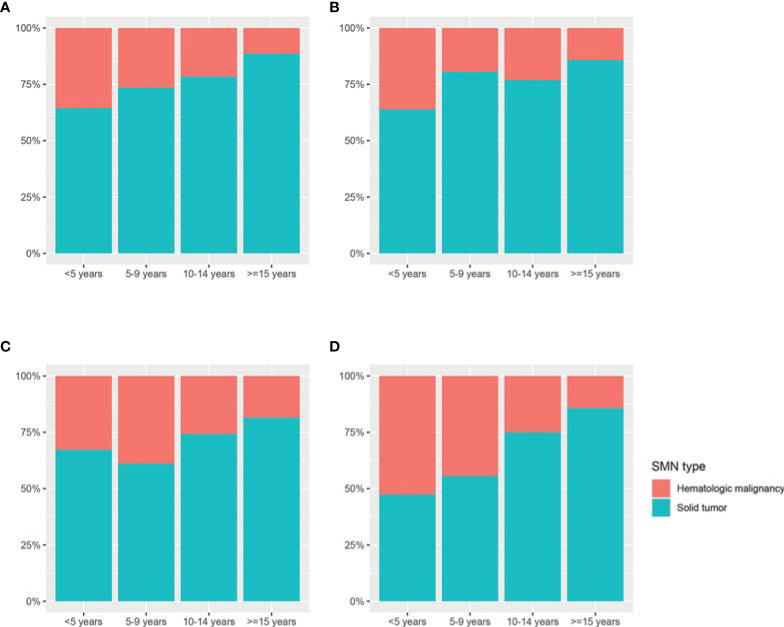
The distribution of second hematologic malignancy and solid tumor in different racial/ethnic groups according to follow-up interval. **(A)** Non-Hispanic whites; **(B)** non-Hispanic blacks; **(C)** Hispanics; and **(D)** Asian/others. *SMN*, second malignant neoplasm.

As shown in **Figure 2**, the proportion of second solid tumors increased with time in non-Hispanic white (Z = 6.68, *p* < 0.001) and Asian/other patients (Z = 2.268, *p* = 0.02), but not in non-Hispanic black and Hispanic patients. Compared with other racial/ethnic groups, Asian/others had the highest proportion of subsequent hematologic malignancy (41.78%) and the lowest proportion of subsequent solid tumors (58.23%) among racial/ethnic subgroups, especially during the first 5 years after Hodgkin lymphoma diagnosis (hematologic malignancy, 52.63%; solid tumor, 47.37%). The composition of second solid tumors varied significantly between races/ethnicities (*p* < 0.001). The proportion of second skin cancer was the highest in non-Hispanic white patients (11.0%), followed by Asian/others (4.3%) and Hispanics (3.3%). Notably, no non-Hispanic black patient developed second skin cancer within the SEER cohort. Moreover, the proportion of SMN in the respiratory system was much higher in non-Hispanic white (16.0%) and non-Hispanic black (18.3%) patients than that in Hispanic (10.7%) and Asian/other patients (6.5%), as shown in **Supplemental Figure 2**.

### Comparison of Risks of SMN and Mortalities Between Races/Ethnicities

**Figure 3:**
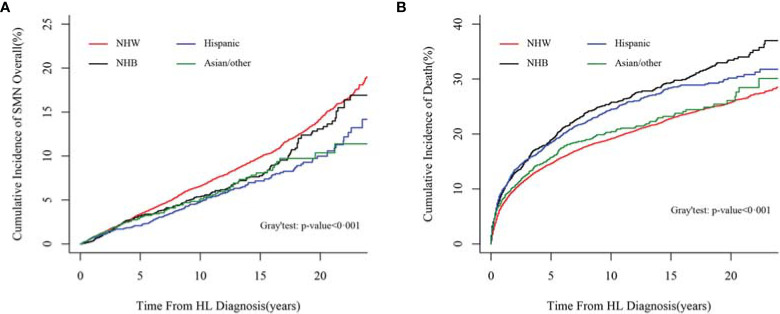
Comparison of cumulative incidences of SMN overall and mortalities between races/ethnicities by the PSH method. **(A)** Comparison of cumulative incidences of SMN overall by the PSH method; **(B)** comparison of cumulative incidences of mortality without SMN by the PSH method. *SMN,* second malignant neoplasm; *PSH*, proportional subdistribution relative hazard.

**Figure 4:**
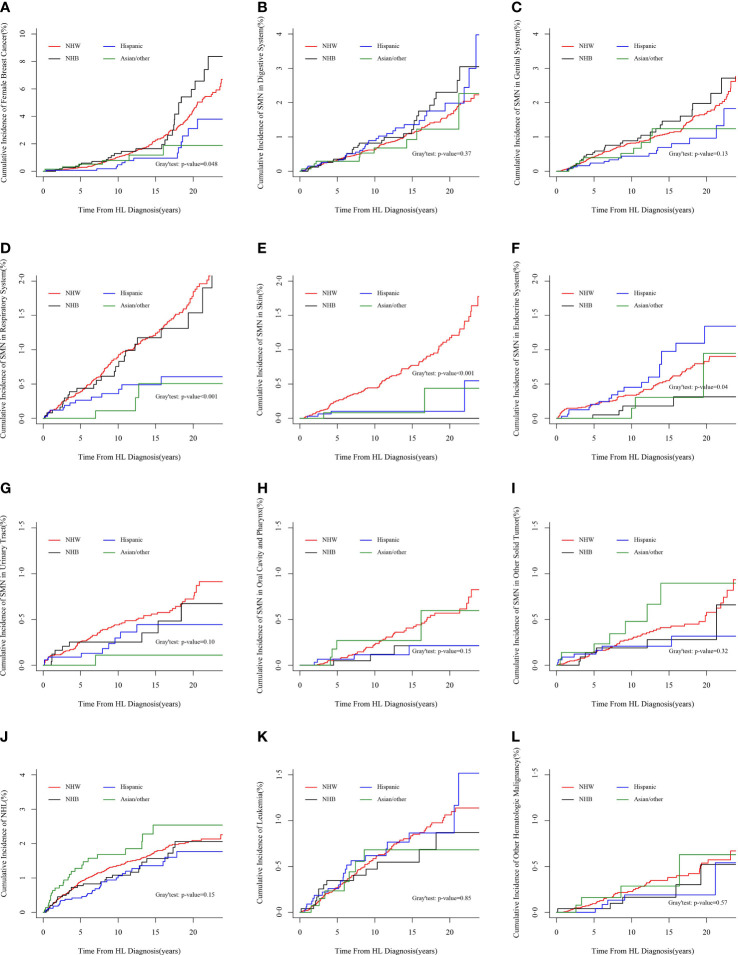
Cumulative incidences of categorized SMN subtypes by PSH method. **(A)** Second female breast cancer, **(B)** SMN in the digestive system, **(C)** SMN in genital system, **(D)** SMN in the respiratory system, **(E)** SMN in the skin, **(F)** SMN in the endocrine system, **(G)** SMN in the urinary system, **(H)** SMN in the oral cavity and pharynx, **(I)** other second solid tumors, **(J)** second NHL, **(K)** second leukemia, and **(L)** other second hematologic malignancy. *SMN*, second malignant neoplasm; *PSH*, proportional subdistribution hazard; *NHL*, non-Hodgkin lymphoma.

The cumulative incidences of SMN were compared among races/ethnicities by PSH and CSH methods, with and without considering competing events. Both methods revealed the racial/ethnic disparities in the incidence of SMN overall (PSH method in **Figure 3A**, and CSH method in **Supplemental Figure 3A**) and specific SMN subtypes (PSH method in **Figure 4** and CSH method in **Supplemental Figure 4** **)**. Both the CSH and PSH regression models were used to assess the effects of race/ethnicity on SMN risk and mortality due to other causes in Hodgkin lymphoma survivors. According to the multicollinearity diagnostic result, there is no multicollinearity between the variables in these regression models (**Supplementary Table 3**).

As shown in **Table 2**, taking non-Hispanic white patients as a reference, non-Hispanic black patients had a 16% overall decreased SMN hazard (PSH, 0.84; 95% CI, 0.72–0.99; *p* = 0.03) in the unadjusted PSH model. After adjusting by age, gender, diagnosis year, stage, and histology subtype, the hazard attenuated statistical insignificance (PSH, 0.86; 95% CI, 0.73–1.01; *p* = 0.06). Additional stratification with the Yost index and treatment did not materially affect results. In the adjusted analysis for SMN subtypes, non-Hispanic black patients demonstrated a 67% relative decreased SMN hazard in the endocrine system (PSH, 0.33; 95% CI, 0.12–0.91; *p* = 0.03). The adjusted hazard ratios for non-Hispanic black patients compared with non-Hispanic white patients for SMN were similar and somewhat greater in the CSH model. Non-Hispanic black female patients had a higher risk for second breast cancer than non-Hispanic white female patients with the use of the CSH method (CSH, 1.56; 95% CI, 1.03–2.36; *p* = 0.04). However, this risk was lower and non-significant with the use of the PSH method (PSH, 1.43; 95% CI, 0.95–2.17; *p* = 0.09) as shown in **Table 3**.

**Table 2:** Cause-specific hazard and proportional subdistribution hazard among Hodgkin lymphoma patients for overall SMN and mortality due to other causes, taking SEER rules for the SMN definition.

	Non-Hispanic Black vs. Non-Hispanic white		Hispanic vs. Non-Hispanic white		Asian/other vs. Non-Hispanic white	
	CSH (95% CI)	PSH (95% CI)	CSH (95% CI)	PSH (95% CI)	CSH (95% CI)	PSH (95% CI)
Model 1: unadjusted						
Death	1.36 (1.25 to 1.48)^*^	1.36 (1.25 to 1.48)^*^	1.26 (1.17 to 1.37)^*^	1.27 (1.17 to 1.37)^*^	1.06 (0.94 to 1.20)^*^	1.06 (0.94 to 1.20)^*^
SMN	0.92 (0.79 to 1.08)	0.84 (0.72 to 0.99)^*^	0.74 (0.64 to 0.87)^*^	0.68 (0.58 to 0.79)^*^	0.77 (0.62 to 0.97)^*^	0.73 (0.58 to 0.92)^*^
Model 2: adjusted for age, gender, diagnosis year, stage, and subtype of HL						
Death	1.44 (1.32 to 1.57)^*^	1.45 (1.33 to 1.58)^*^	1.35 (1.25 to 1.46)^*^	1.36 (1.25 to 1.47)^*^	1.28 (1.13 to 1.44)^*^	1.26 (1.12 to 1.43)^*^
SMN	0.94 (0.80 to 1.10)	0.86 (0.73 to 1.01)	0.84 (0.71 to 0.98)^*^	0.73 (0.63 to 0.86)^*^	0.89 (0.71 to 1.11)	0.80 (0.64 to 1.01)
Model 3: additionally adjusted for Yost index						
Mortality	1.43 (1.31 to 1.46)^*^	1.44 (1.31 to 1.57)^*^	1.34 (1.24 to 1.45)^*^	1.34 (1.24 to 1.46)^*^	1.29 (1.14 to 1.46)^*^	1.28 (1.13 to 1.45)^*^
SMN	0.93 (0.79 to 1.09)	0.86 (0.73 to 1.01)	0.83 (0.71 to 0.97)^*^	0.73 (0.62 to 0.86)^*^	0.89 (0.71 to 1.12)	0.80 (0.64 to 1.01)
Model 4: additionally adjusted for chemotherapy and radiotherapy						
Mortality	1.35 (1.24 to 1.47)^*^	1.36 (1.24 to 1.49)^*^	1.30 (1.20 to 1.40)^*^	1.30 (1.20 to 1.41)^*^	1.29 (1.15 to 1.46)^*^	1.28 (1.13 to 1.46)^*^
SMN	0.93 (0.79 to 1.09)	0.88 (0.75 to 1.03)	0.83 (0.71 to 0.98)^*^	0.74 (0.63 to 0.87)^*^	0.89 (0.71 to 1.12)	0.80 (0.64 to 1.01)

^*^p < 0.05.

An SMN diagnosis was assigned to patients who developed a malignancy at least 2 months after the index Hodgkin lymphoma diagnosis according to the criteria for multiple primary cancers developed by the SEER program.

SMN, second malignant neoplasm; SEER, Surveillance, Epidemiology, and End Results.

**Table 3:** Cause-specific hazard and proportional subdistribution hazard among Hodgkin lymphoma patients for categorized SMN subtypes, taking SEER rules for the SMN definition.

	Non-Hispanic Black vs. Non-Hispanic white		Hispanic vs. Non-Hispanic white		Asian/other vs. Non-Hispanic white	
	CSH (95% CI)	PSH (95% CI)	CSH (95% CI)	PSH (95% CI)	CSH (95% CI)	PSH (95% CI)
Skin excluding basal and squamous	NA	NA	0.24 (0.09 to 0.65)^*^	0.22 (0.08 to 0.59)^*^	0.26 (0.06 to 1.04)	0.23 (0.06 to 0.93)^*^
Oral cavity and pharynx	0.43 (0.13 to 1.39)	0.40 (0.12 to 1.29)	0.49 (0.18 to 1.35)	0.43 (0.15 to 1.20)	1.21 (0.44 to 3.36)	1.07 (0.38 to 2.98)
Digestive system	1.45 (0.97 to 2.17)	1.38 (0.92 to 2.08)	1.51 (1.04 to 2.21)^*^	1.34 (0.91 to 1.96)	1.10 (0.58 to 2.09)	0.97 (0.51 to 1.86)
Female breast	1.56 (1.03 to 2.36)^*^	1.43 (0.95 to 2.17)	0.62 (0.35 to 1.10)	0.55 (0.31 to 0.98)^*^	0.62 (0.27 to 1.40)	0.55 (0.25 to 1.24)
Respiratory system	1.09 (0.71 to 1.69)	1.04 (0.67 to 1.62)	0.52 (0.29 to 0.91)^*^	0.45 (0.26 to 0.79)^*^	0.30 (0.09 to 0.93)^*^	0.26 (0.08 to 0.82)^*^
Genital system	1.33 (0.89 to 1.99)	1.26 (0.85 to 1.88)	0.73 (0.45 to 1.19)	0.65 (0.40 to 1.07)	0.97 (0.51 to 1.85)	0.87 (0.46 to 1.64)
Urinary system	0.83 (0.41 to 1.66)	0.80 (0.39 to 1.62)	0.74 (0.37 to 1.49)	0.68 (0.34 to 1.38)	0.20 (0.03 to 1.43)	0.18 (0.03 to 1.30)
Endocrine system	0.35 (0.13 to 0.95)^*^	0.33 (0.12 to 0.91)^*^	1.31 (0.78 to 2.17)	1.24 (0.75 to 2.07)	0.50 (0.16 to 1.59)	0.47 (0.15 to 1.53)
Other solid tumor	0.79 (0.34 to 1.86)	0.74 (0.32 to 1.71)	0.78 (0.35 to 1.73)	0.72 (0.33 to 1.61)	1.86 (0.84 to 4.10)	1.75 (0.81 to 3.78)
NHL	0.81 (0.55 to 1.19)	0.78 (0.54 to 1.14)	0.84 (0.58 to 1.22)	0.77 (0.53 to 1.12)	1.59 (1.05 to 2.41)^*^	1.48 (0.98 to 2.25)
Leukemia	0.93 (0.52 to 1.66)	0.89 (0.50 to 1.59)	1.21 (0.75 to 1.94)	1.10 (0.67 to 1.78)	0.98 (0.45 to 2.11)	0.90 (0.42 to 1.92)
Other hematologic malignancy	0.75 (0.29 to 1.90)	0.72 (0.28 to 1.83)	0.70 (0.28 to 1.76)	0.62 (0.25 to 1.55)	1.30 (0.47 to 3.62)	1.15 (0.42 to 3.19)

^*^p < 0.05.

An SMN diagnosis was assigned to patients who developed a malignancy at least 2 months after the index Hodgkin lymphoma diagnosis according to the criteria for multiple primary cancers developed by SEER program. All these hazards were adjusted by age, gender, diagnosis year of Hodgkin lymphoma, Ann Arbor stage, histology, Yost index, and treatment as appropriate.

SMN, second malignant neoplasm; NHL, non-Hodgkin lymphoma; SEER, Surveillance, Epidemiology, and End Results. NA, not available.

Hispanic patients had a 32% decreased SMN hazard overall (PSH, 0.68; 95% CI, 0.58–0.79; *p* < 0.001) than non-Hispanic white patients in the unadjusted PSH analysis. After adjusting by age, gender, diagnosis year, stage, and histology, the hazard increased to 0.73 (95% CI, 0.63–0.86; *p* < 0.001). Additional stratification with the Yost index yields a similar result. After additional treatment adjustment, the hazard increased further to 0.74 (95% CI, 0.63–0.87; *p* < 0.001). In the adjusted analysis for SMN subtypes, Hispanic patients demonstrated a 78% relative decreased hazard of subsequent skin cancer (PSH, 0.22; 95% CI, 0.08–0.59; *p* = 0.02) and a 55% decreased SMN hazard in the respiratory system (PSH, 0.45; 95% CI, 0.26–0.79; *p* = 0.04). Again, the CSH method yields similar but somewhat higher hazards. Hispanic patients had a higher SMN risk in the digestive system than non-Hispanic white patients with the use of the CSH method (CSH, 1.51; 95% CI, 1.04–2.21; *p* = 0.03). This risk was lower and non-significant with the use of the PSH method (PSH, 1.34; 95% CI, 0.91–1.96; *p* = 0.13), as shown in **Table 3**.

Asian/other patients had a 27% decreased overall SMN hazard (PSH, 0.73; 95% CI, 0.58–0.92; *p* < 0.001) than non-Hispanic white patients in the unadjusted PSH analysis. After adjusting by age, gender, diagnosis year, stage, and histology subtype, the hazard increased to 0.80 (95% CI, 0.64–1.01; *p* = 0.06). Additional stratification with the Yost index and treatment yields similar results. In the adjusted analysis for SMN subtypes, Asian/other patients demonstrated a 74% relative decreased PSH of SMN in the respiratory system (PSH, 0.26; 95% CI, 0.08–0.82; *p* = 0.02) and a 77% relative decreased PSH of subsequent skin cancer (PSH, 0.23; 95% CI, 0.06–0.93, *p* = 0.04). The CSH method yields results that differed somewhat from the PSH method. Asian/other patients had a higher risk for subsequent non-Hodgkin lymphoma (NHL) than non-Hispanic white patients with the use of the CSH method (CSH, 1.59; 95% CI, 1.05–2.41; *p* = 0.03). However, this risk was lower and non-significant with the use of the PSH method (PSH, 1.48; 95% CI, 0.98–2.25; *p* = 0.06), as shown in **Table 3**.

The cumulative incidences of mortality before experiencing a second cancer were compared among different racial/ethnic groups by the PSH and CSH methods (PSH method in **Figure 3B** and CSH method in **Supplemental Figure 3B**). Non-Hispanic white patients were less likely to experience a mortality event before developing SMN than the other three groups. In the fully adjusted model, taking non-Hispanic whites as a reference, the PSH for NHB, Hispanic, and Asian/other patients was 1.36 (95% CI, 1.24–1.49; *p* < 0.001), 1.30 (95% CI, 1.20–1.41; *p* < 0.001), and 1.28 (95% CI, 1.13–1.46; *p* < 0.001), respectively. The results from the CSH model were similar (**Table 2**).

### Sensitivity Analyses

These findings above were similar in the sensitivity analyses of the present study to exclude SMN diagnosed within 6 months of the primary Hodgkin lymphoma (**Supplemental Tables 4** and **5**). Indeed, the most significant difference observed was the SMN risk in the endocrine system. With the SEER criteria, both PSH and CSH methods showed that non-Hispanic black patients had a significantly lower SMN hazard in the endocrine system when compared with non-Hispanic white patients (**Table 3**). However, the hazard was higher and attenuated statistical insignificance by both methods using the IACR/IARC criteria.

## Discussion

To obtain a dynamic understanding of the racial/ethnic effects on SMN among Hodgkin lymphoma survivors, the PSH and the CSH methods were used in the present study. Both methods showed that, compared with non-Hispanic white patients, non-Hispanic patients had a lower SMN risk in the endocrine system; Hispanic patients had a lower risk for SMN overall, SMN in the respiratory system, and SMN in the skin; and Asian/others had a lower risk for SMNs in the respiratory system. Some differences were also found between the PSH and the CSH results. For instance, CSH analysis showed that Asian/other patients had no significantly lower risk for subsequent skin cancer, but the risk decreased further and became statistically significant with the PSH method. The differences observed between the two methods highlight the differing interpretations of both utilities for understanding the racial/ethnic effects on SMN in Hodgkin lymphoma survivors. CSH shows whether race/ethnicity is directly associated with SMN risk in Hodgkin lymphoma survivors without considering the competing events. However, the PSH method shows whether race/ethnicity affects the actual probability of experiencing second cancer regardless of the direct association.

In the competing risk analysis of the present study, the cumulative mortality due to other causes was found to be lower in non-Hispanic white and Asian/other patients and higher in Hispanic and non-Hispanic black patients. Consistent with existing literature, non-Hispanic black and Hispanic patients with Hodgkin lymphoma tend to have a worse outcome. A population-based analysis has shown that the 5-year overall survival rates for non-Hispanic black (76%) and Hispanic (75%) patients were lower compared with non-Hispanic whites (82%) and non-Hispanic Asians (81%) (18). Among children with Hodgkin lymphoma, Hispanic and non-Hispanic black children demonstrated a higher hazard of post-relapse mortality than non-Hispanic black children (24). Moreover, the adjusted hazard from both methods in the present study suggested that Asian/other patients also had a higher risk of mortality due to other causes than non-Hispanic patients.

Previous studies have shown an increased SMN risk among Hodgkin lymphoma survivors (7, 13, 14, 29, 40, 41). However, these studies were mainly based on white cohorts, and information on other races was limited. Lisa et al. recently noted that the Asian race was associated with SMN risk (42). However, in the present population-based cohort, Asian/other patients were shown to increase the risk of subsequent NHL, but not SMN overall. The different observation with the prior study may be caused by different inclusion criteria and conception of race.

This study is believed to be the first study to comprehensively evaluate the association between race/ethnicity and SMN among Hodgkin lymphoma survivors. This report suggested that SMN rate is lowest in Hispanic patients, and mortality due to other causes is lowest in non-Hispanic white patients. For non-Hispanic black patients, both SMN rate and mortality due to other causes are relatively high. Asian/other patients have a relatively low cumulative SMN incidence and mortality due to other causes and show a different SMN distribution when compared with other racial/ethnic groups. Asian/others have the highest proportion of subsequent hematologic malignancy and seem to more likely develop NHL than other groups. All the aforementioned suggested that race/ethnicity should be considered when developing strategies for survivorship care among Hodgkin lymphoma survivors. It is worth noting that the racial/ethnic impact pattern on SMN risk could differs between Hodgkin lymphoma and all cancer survivors. A large cohort study that included young patients diagnosed with invasive cancer between 1990 and 2012 has revealed that, compared with non-Hispanic white patients, Asian/Pacific Islanders were associated with a lower risk for SMN overall, but Hispanics were not (43).

Parsing out the underlying cause for the association between race/ethnicity and SMN in the present study is challenging. The proposed hypotheses for cancer health disparities often relate to racial/ethnic differences in host biology or differences in socioeconomic status and healthcare access (44). In the present study, differences in SMN risk may not entirely be explained by socioeconomic status and treatment because the adjustment for Yost index and treatment type did not change the results. Genetic or biological attributes in each race/ethnicity group may explain the observed distribution of SMN risk in the present study. However, no relevant research was noted on racial differences in genetic factors associated with Hodgkin lymphoma. Besides genetic factors, differences in lifestyles may be a possible explanation for this observation. Lung cancers, as first or second neoplasm, are well-known to be influenced by smoking histories (45, 46). Interestingly, previous studies have reported that individuals of white and black descent have a higher smoking prevalence than Asians and Hispanics (21), which may take partial part in the higher second lung cancer incidence in non-Hispanic white and non-Hispanic black patients with Hodgkin lymphoma. Physical inactivity, excess body weight, and some aspects of the Western diet are known risk factors for colon cancer (47–49). Previous studies had reported that Hispanics were engaged in less healthy exercise and dietary behaviors than non-Hispanic whites (50–52). In the present study, we also identified that Hispanics had a higher risk for subsequent colon cancer when compared with non-Hispanic whites. Interventions focused on these factors may reduce racial/ethnic differences in certain second cancer incidence.

The present study includes a large number of Hodgkin lymphoma survivors from a population-based setting, which eliminated biases in hospital-based series. The present study also has some limitations. Some variables that would also potentially influence the risk of SMNs in Hodgkin lymphoma survivors (e.g., family history, genetic information, and lifestyle characteristics) were unavailable. Some information about treatment is missing out, and the SEER dataset only collects the initial treatment type; the detailed drugs, doses, radiation fields, and subsequent therapy patients received are unknown, potentially biasing the results. Moreover, there were no uniform criteria for SMN. The criteria for defining SMN differ between studies; analysis with different definitions may yield different results. However, the impact seems not large, based on the sensitivity analysis. An additional limitation is the multiple comparisons without correction that we undertook, given the exploratory nature of this study. Further research to validate the association between race/ethnicity and SMN among Hodgkin lymphoma survivors is needed.

## Conclusions

In summary, the findings of the present study revealed substantial racial/ethnic differences in the SMN risk and mortality among Hodgkin lymphoma patients. The dual analysis with CSH and PSH methods provides a comprehensive view of racial/ethnic effects on SMN risk among Hodgkin lymphoma survivors. These findings suggest that race/ethnicity needs to be considered in future cancer surveillance for patients with Hodgkin lymphoma.

## Data Availability Statement

Publicly available datasets were analyzed in this study. These data can be found here: researchers interested in the SEER database may submit an inquiry online, at https://seer.cancer.gov/data/access.html.

## Ethics Statement

Ethical review and approval were not required for the study on human participants in accordance with the local legislation and institutional requirements. Written informed consent from the participants’ legal guardian/next of kin was not required to participate in this study in accordance with the national legislation and the institutional requirements.

## Author Contributions

LW, QX, and HX contributed to the conception and design of the study. LW, HX, JH, and SL contributed to data curation. LW, HX, JH, DC, and QZ performed the statistical analysis. HX, LL, XY and JC wrote the first draft of the manuscript. HX wrote the first draft of the manuscript. All authors contributed to the article and approved the submitted version.

## Funding

The present study was supported by grants from Chongqing Science and Health Joint Project [2018ZDXM001], Chongqing Science and Technology Commission [cstc2018jcyjAX0688], Chongqing Municipal Education Commission [KJ1702017], and Science and Technology Planning Project of Yuzhong District of Chongqing [20190121]. None of the funders had a role in the design and conduct of the study; in the collection, management, analysis, and interpretation of the data; in the preparation, review, or approval of the manuscript; or in the decision to submit the manuscript for publication.

## Conflict of Interest

The authors declare that the research was conducted in the absence of any commercial or financial relationships that could be construed as a potential conflict of interest.

## Publisher’s Note

All claims expressed in this article are solely those of the authors and do not necessarily represent those of their affiliated organizations, or those of the publisher, the editors and the reviewers. Any product that may be evaluated in this article, or claim that may be made by its manufacturer, is not guaranteed or endorsed by the publisher.
