# Supplementary material for: Racial/Ethnic Disparities on the Risk of Second Malignant Neoplasm Among Hodgkin Lymphoma Survivors
Source: Front Oncol. 2022 Jan 24;11:790891. doi: 10.3389/fonc.2021.790891 (PMC8818723; doi:10.3389/fonc.2021.790891)

Supplementary Material

**Supplemental Table 1.** Summary of how SMNs were re-categorized into twelve different subtypes.

| **Categories** | **ICD-O-3 Site** | **ICD-O-3 Histology (Type)** |
| --- | --- | --- |
| Female breast | C500-C509 (female) | excluding 9050-9055, 9140, 9590-9992 |
| Female Genital System | C530-C539, C540-C549, C559, C569, C529, C510-C519, C570-C579, C589 | excluding 9050-9055, 9140, 9590-9992 |
| Male Genital System | C619, C620-C629, C600-C609, C630-C639 | excluding 9050-9055, 9140, 9590-9992 |
| Digestive System | C150-C189, C260, C199, C209, C210-C212, C218, C220, C221, C239, C240C259, C480 -C482, C268-C269, C488 | excluding 9050-9055, 9140, 9590-9992 |
| Oral Cavity and Pharynx | C000-C009, C019-C029, C079-C089, C040-C049, C030-C039, C050-C059, C060-C069, C110-C119, C090-C099, C100-C109, C129, C130-C139, C140, C142, C148 | excluding 9050-9055, 9140, 9590-9992 |
| Respiratory System | C300-C301, C310-C319, C320-C329, C340-C349, C339, C381-C383, C388, C390, C398, C399 | excluding 9050-9055, 9140, 9590-9992 |
| Skin excluding Basal and Squamous | C440-C449, C440-C449 | excluding 8000-8005, 8010-8046, 8050-8084, 8090-8110, 9050-9055, 9140, 9590-9992 |
| Urinary system | C670-C679, C649, C659, C669, C680-C689 | excluding 9050-9055, 9140, 9590-9992 |
| Other solid tumors | C400-C419, C380, C470-C479, C490-C499, C500-C509(male), C690-C699, C710-C719, C700-C709, C720-C729, C500-C509 (male) | excluding 9050-9055, 9140, 9590-9992 |
| NHL |  | 9590-9597, 9670-9671, 9673, 9675, 9678-9680, 9684, 9687-9691, 9695, 9698-9702, 9705, 9708-9709, 9712, 9714-9719, 9724-9729, 9735, 9737-9738, 9811-9818, 9823, 9827, 9837 |
| Leukemia |  | 9826, 9835-9836, 9811-9818, 9837, 9823, 9820, 9832-9834, 9940, 9840, 9861, 9865-9867, 9869, 9871-9874, 9895-9897, 9898, 9910-9911, 9920, 9891, 9863, 9875-9876, 9945-9946, 9860, 9930, 9801, 9805-9809, 9931, 9733, 9742, 9800, 9831, 9870, 9948, 9963-9964, 9827 |
| Other hematologic malignancy |  | 9740-9741, 9750-9769, 9950, 9960-9962, 9965-9967, 9970-9971, 9975, 9980, 9982-9987, 9989, 9991-9992, 9731-9732, 9734 |

# *ICD-O-3* the International Classification of Diseases for Oncology, third edition; *SMN* second malignant neoplasm; *NHL* Non-Hodgkin lymphoma.

**Supplemental Table 2.** Event numbers in patients with primary Hodgkin lymphoma.

|  | **non-Hispanic White**  (n=15025) | **non-Hispanic Black**  (n=2513) | **Hispanic**  (n=3424) | **Asian/other**  (n=1453) |
| --- | --- | --- | --- | --- |
| Events |  |  |  |  |
| Death without SMN | 3078(20.49%) | 641(25.51%) | 773(22.58%) | 282(19.41%) |
| SMN | 1347(8.97%) | 176(7.00%) | 176(5.14%) | 79(5.44%) |
| Solid tumor |  |  |  |  |
| Skin excluding Basal and Squamous | 110(0.73%) | 0(0.00%) | 4(0.12%) | 2(0.14%) |
| Oral Cavity and Pharynx | 53(0.35%) | 3(0.12%) | 4(0.12%) | 4(0.28%) |
| Digestive system | 150(1.00%) | 29(0.19%) | 34(0.99%) | 10(0.69%) |
| Female Breast | 155(1.03%) | 27(1.07%) | 13(0.38%) | 6(0.41%) |
| Respiratory System | 160(1.06%) | 24(0.96%) | 13(0.38%) | 3(0.21%) |
| Genital System | 165(1.10%) | 29(1.15%) | 18(0.53%) | 10(0.69%) |
| Urinary system | 77(0.51%) | 9(0.36%) | 9(0.26%) | 1(0.07%) |
| Endocrine System | 75(0.50%) | 4(0.16%) | 19(0.55%) | 3(0.21%) |
| Other solid tumor | 57(0.38%) | 6(0.24%) | 7(0.82%) | 7(1,93%) |
| Hematologic malignancy |  |  |  |  |
| NHL | 223(1.48%) | 31(1.23%) | 33(0.94%) | 25(1.72%) |
| Leukemia | 73(0.49%) | 9(0.36%) | 17(0.50%) | 4(0.28%) |
| Other hematologic malignancy | 49(0.33%) | 5(0.20%) | 5(0.15%) | 4(0.28%) |
| No events | 10600(70.55%) | 1696(67.49%) | 2475(72.28%) | 1092(75.15%) |

# *SMN* second malignant neoplasm, *NHL* Non-Hodgkin lymphoma.

**Supplementary Table 3**. Variance inflation factors of included variables in regression models.

|  | SMN | | Death due to other causes | |
| --- | --- | --- | --- | --- |
|  | CSH | PSH | CSH | PSH |
| Age | 1.02 | 1.05 | 1.02 | 1.02 |
| Yost index | 1.02 | 1.05 | 1.03 | 1.04 |
| Year of diagnosis | 1.03 | 1.06 | 1.05 | 1.08 |
| Sex | 1.01 | 1.02 | 1.01 | 1.03 |
| Radiotherapy | 1.17 | 1.21 | 1.16 | 1.18 |
| Chemotherapy | 1.11 | 1.06 | 1.09 | 1.20 |
| Histology subtype | 1.06 | 1.08 | 1.02 | 1.04 |
| Ann Arbor stage | 1.20 | 1.24 | 1.20 | 1.29 |
| Non-Hispanic White (Ref) | - | - |  | - |
| Non-Hispanic Black | 1.05 | 1.06 | 1.07 | 1.07 |
| Hispanic | 1.03 | 1.06 | 1.07 | 1.03 |
| Asian/other | 1.02 | 1.02 | 1.03 | 1.07 |

The “rms” package in R was used to calculate variance inflation factors.

Abbreviations: *SMN* second malignant neoplasm; *CSH* Cause-specific hazard; *PSH* Proportional subdistribution hazard.

**Supplemental Table 4.** Cause-specific hazard and proportional subdistribution hazard among Hodgkin lymphoma patients for SMN overall and mortality without SMN (taking non-Hispanic whites as reference).

|  | **non-Hispanic Black** | | **Hispanic** | | **Asian/other** | |
| --- | --- | --- | --- | --- | --- | --- |
|  | CSH (95%CI) | PSH (95%CI) | CSH (95%CI) | PSH (95%CI) | CSH (95%CI) | PSH (95%CI) |
| Model 1: unadjusted |  |  |  |  |  |  |
| Death | 1.36(1.25-1.48) ^*^ | 1.35(1.24-1.47) ^*^ | 1.26(1.17-1.37) ^*^ | 1.27(1.17-1.37) ^*^ | 1.06(0.94-1.20) ^*^ | 1.06(0.94-1.20) ^*^ |
| SMN | 0.94(0.80-1.11) | 0.86(0.73-1.01) | 0.74(0.63-0.86) ^*^ | 0.67(0.57-0.78) ^*^ | 0.76(0.60-0.96) ^*^ | 0.72(0.57-0.91) ^*^ |
| Model 2: adjusted for age, sex, diagnosis year, stage and subtype of HL |  |  |  |  |  |  |
| Death | 1.44(1.32-1.57) ^*^ | 1.45(1.32-1.58) ^*^ | 1.35(1.25-1.46) ^*^ | 1.36(1.25-1.47) ^*^ | 1.28(1.13-1.44) ^*^ | 1.27(1.12-1.44) ^*^ |
| SMN | 0.96(0.81-1.12) | 0.88(0.75-1.03) | 0.83(0.70-0.97) ^*^ | 0.72(0.62-0.85) ^*^ | 0.96(0.81-1.12) | 0.79(0.62-1.00) |
| Model 3: additionally adjusted for Yost index |  |  |  |  |  |  |
| Death | 1.43(1.31-1.55) ^*^ | 1.43(1.31-1.56) ^*^ | 1.34(1.24-1.45) ^*^ | 1.35(1.24-1.46) ^*^ | 1.29(1.14-1.46) ^*^ | 1.29(1.14-1.46) ^*^ |
| SMN | 0.95(0.81-1.12) | 0.88(0.75-1.03) | 0.82(0.70-0.97) ^*^ | 0.72(0.61-0.85) ^*^ | 0.88(0.69-1.11) | 0.79(0.62-1.00) |
| Model 4: additionally adjusted for chemotherapy and radiotherapy |  |  |  |  |  |  |
| Death | 1.35(1.15-1.46) ^*^ | 1.36(1.24-1.49) ^*^ | 1.30(1.20-1.40) ^*^ | 1.31(1.20-1.42) ^*^ | 1.30(1.15-1.46) ^*^ | 1.29(1.14-1.46) ^*^ |
| SMN | 0.95(0.81-1.12) | 0.90(0.76-1.05) | 0.83(0.70-0.98) ^*^ | 0.73(0.62-0.87) ^*^ | 0.88(0.69-1.11) | 0.79(0.62-1.00) |

^*^*p* < 0.05

An SMN diagnosis was assigned to patients who developed a malignancy at least six months after the index HL diagnosis according to the criteria for multiple primary cancers developed by IACR/IARC. *SMN* second malignant neoplasm.

**Supplemental Table 5.** Cause-specific hazard and proportional subdistribution hazard among Hodgkin lymphoma patients for categorized SMN subtypes (taking non-Hispanic whites as a reference).

|  | non-Hispanic Black | | Hispanic | | Asian/other | |
| --- | --- | --- | --- | --- | --- | --- |
|  | CSH (95%CI) | PSH (95%CI) | CSH (95%CI) | PSH (95%CI) | CSH (95%CI) | PSH (95%CI) |
| Skin excluding Basal and Squamous | NA | NA | 0.24(0.09-0.65) ^*^ | 0.22(0.08-0.59) ^*^ | 0.26(0.06-1.04) | 0.23(0.06-0.93) ^*^ |
| Oral Cavity and Pharynx | 0.42(0.13-1.39) | 0.39(0.12-1.29) | 0.49(0.18-1.35) | 0.43(0.15-1.21) | 1.21(0.44-3.36) | 1.07(0.39-2.98) |
| Digestive system | 1.49(1.00-2.24) | 1.42(0.94-2.13) | 1.52(1.04-2.23) ^*^ | 1.35(0.91-1.98) | 1.01(0.51-1.98) | 0.90(0.46-1.76) |
| Female Breast | 1.52(1.00-2.31) ^*^ | 1.42(0.93-2.16) | 0.63(0.35-1.13) | 0.54(0.30-0.97) ^*^ | 0.50(0.20-1.22) | 0.46(0.19-1.11) |
| Respiratory System | 1.06(0.68-1.67) | 1.01(0.64-1.59) | 0.51(0.28-0.92) ^*^ | 0.45(0.25-0.80) ^*^ | 0.32(0.10-0.99) ^*^ | 0.28(0.09-0.88) ^*^ |
| Genital System | 1.34(0.90-2.01) | 1.27(0.85-1.90) | 0.73(0.45-1.20) | 0.66(0.40-1.08) | 0.98(0.52-1.86) | 0.88(0.46-1.65) |
| Urinary system | 0.92(0.46-1.87) | 0.89(0.43-1.82) | 0.67(0.30-1.46) | 0.60(0.27-1.33) | 0.23(0.03-1.64) | 0.21(0.03-1.48) |
| Endocrine System | 0.41(0.15-1.13) | 0.38(0.14-1.07) | 1.58(0.94-2.65) | 1.50(0.90-2.52) | 0.60(0.19-1.92) | 0.57(0.18-1.85) |
| Other solid tumor | 0.81(0.34-1.90) | 0.76(0.33-1.75) | 0.59(0.23-1.47) | 0.54(0.21-1.36) | 1.71(0.73-4.01) | 1.65(0.71-3.82) |
| NHL | 0.84(0.57-1.24) | 0.81(0.55-1.18) | 0.82(0.56-1.21) | 0.75(0.50-1.10) | 1.56(1.01-2.41) ^*^ | 1.45(0.94-2.24) |
| Leukemia | 0.89(0.48-1.62) | 0.85(0.46-1.55) | 1.24(0.77-2.00) | 1.14(0.71-1.84) | 1.01(0.47-2.18) | 0.93(0.43-1.99) |
| Other hematologic malignancy | 0.60(0.21-1.67) | 0.57(0.20-1.62) | 0.70(0.28-1.77) | 0.62(0.25-1.56) | 1.31(0.47-3.65) | 1.17(0.42-3.24) |

^*^*p* < 0.05. All these hazards were adjusted by age, sex, diagnosis year of Hodgkin lymphoma, Ann Arbor stage, histology, Yost index, and treatment when appropriate.

An SMN diagnosis was assigned to patients who developed a malignancy at least six months after the index HL diagnosis according to the criteria for multiple primary cancers developed by IACR/IARC. *SMN* second malignant neoplasm, *NHL* non-Hodgkin lymphoma.

**Supplemental Figure 1. Cumulative incidence of SMN overall and mortalities after the primary Hodgkin lymphoma diagnosis.** (A) non-Hispanic whites; (B) non-Hispanic blacks; (C) Hispanics; (D) Asian/others. *SMN* second malignant neoplasm. *HL* Hodgkin lymphoma.

**
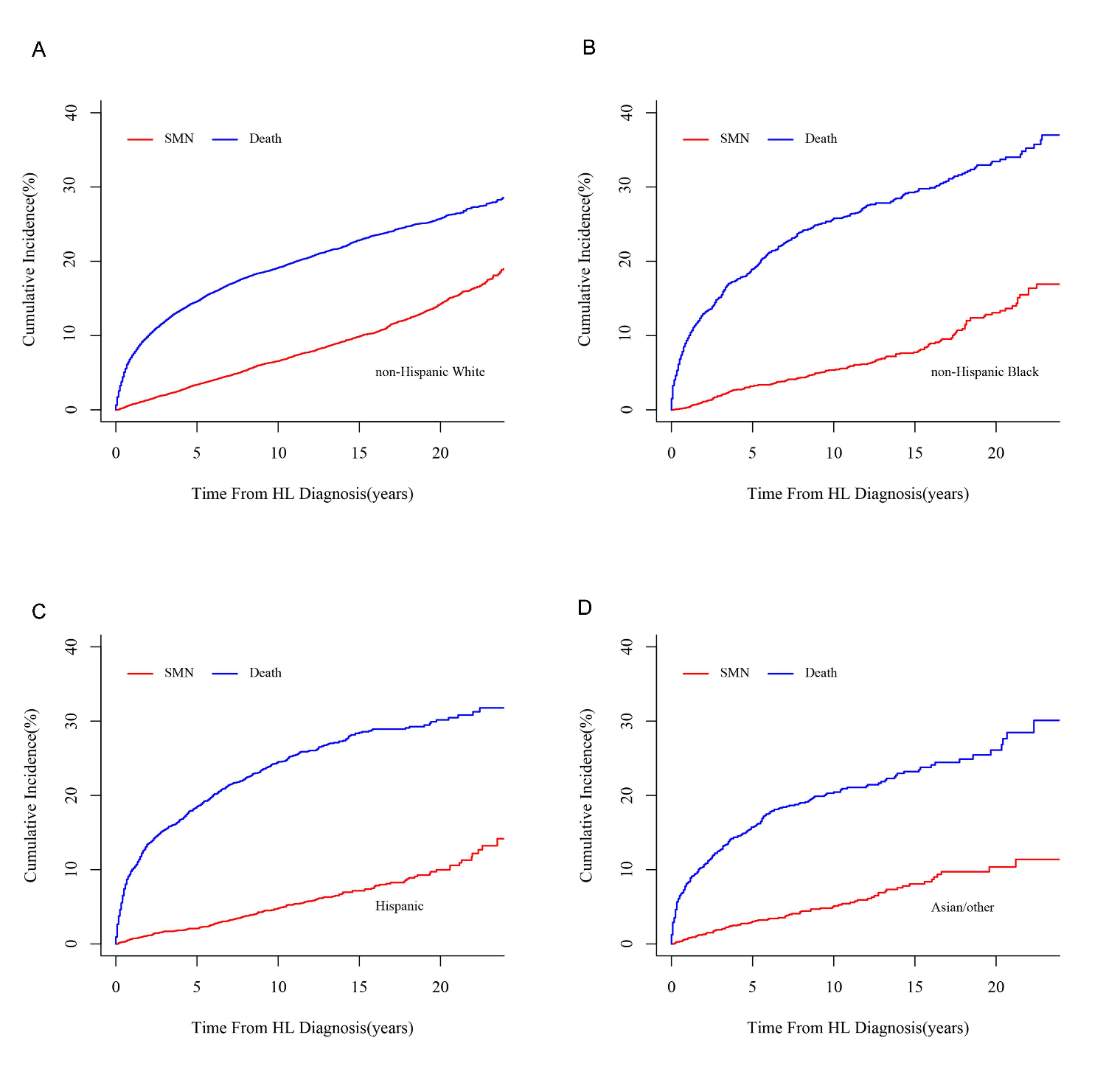
**

**Supplemental Figure 2. The distribution of specific SMN subtypes in different racial/ethnic groups.** (A) The distribution of second solid tumors in non-Hispanic whites; (B) the distribution of second hematologic malignancy in non-Hispanic whites; (C) the distribution of second solid tumors in non-Hispanic blacks; (D) the distribution of second hematologic malignancy in non-Hispanic blacks; (E) the distribution of second solid tumors in Hispanics; (F) the distribution of second hematologic malignancy in Hispanics; (G) the distribution of second solid tumors in Asian/others; and (H) the distribution of second hematologic malignancy in Asian/others.
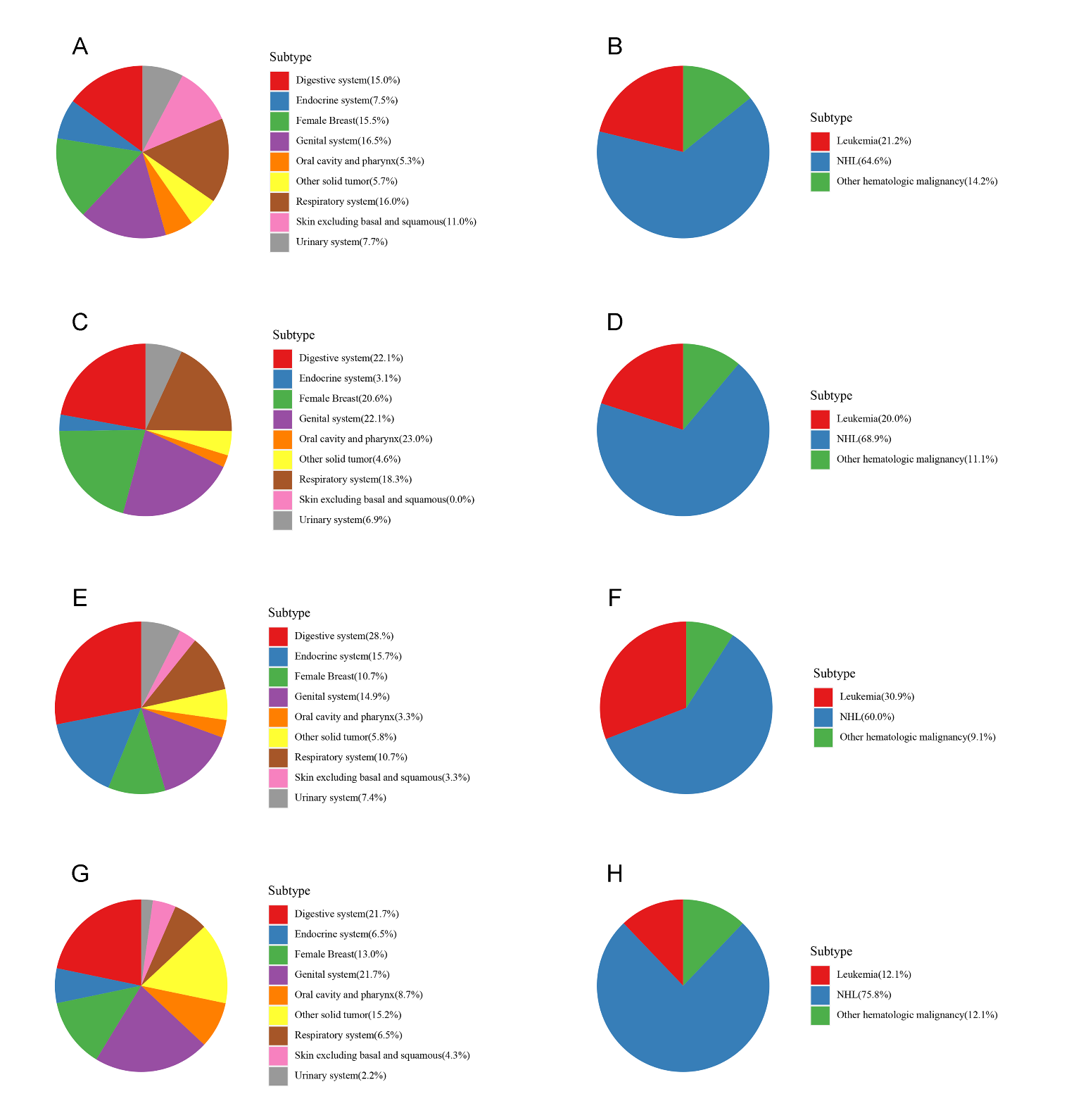


**Supplemental Figure 3. Comparison of cumulative incidences of SMN overall and mortalities between races/ethnicities.** (A) Comparison of cumulative incidences of SMN overall by CSH method; (B) comparison of cumulative incidences of mortality without SMN by CSH method. *CSH* cause-specific hazard, *SMN* second malignant neoplasm.


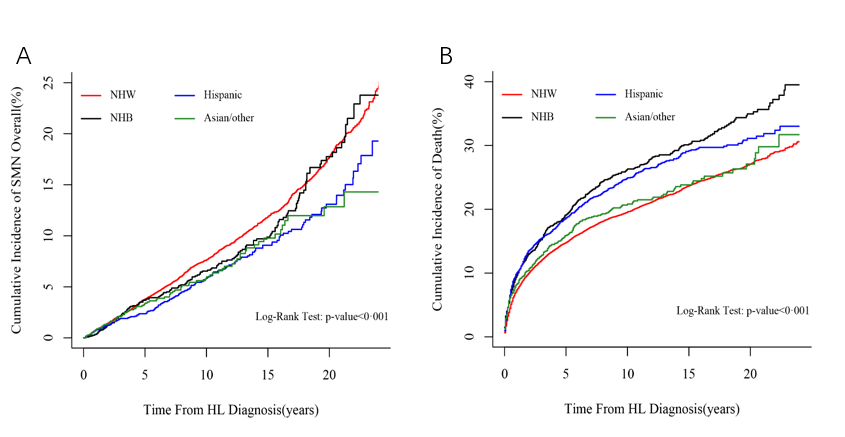


**Supplemental Figure 4. Comparison of cumulative incidences of SMN overall and mortalities between races/ethnicities.** (A) Comparison of cumulative incidences of SMN overall by PSH method; (B) comparison of cumulative incidences of overall SMN by the CSH method; (C) comparison of cumulative incidences of mortality without SMN by PSH method; (D) comparison of cumulative incidences of mortalities without SMN by CSH method. *SMN* second malignant neoplasm, *PSH* proportional subdistribution relative hazard, *CSH* cause-specific hazard.


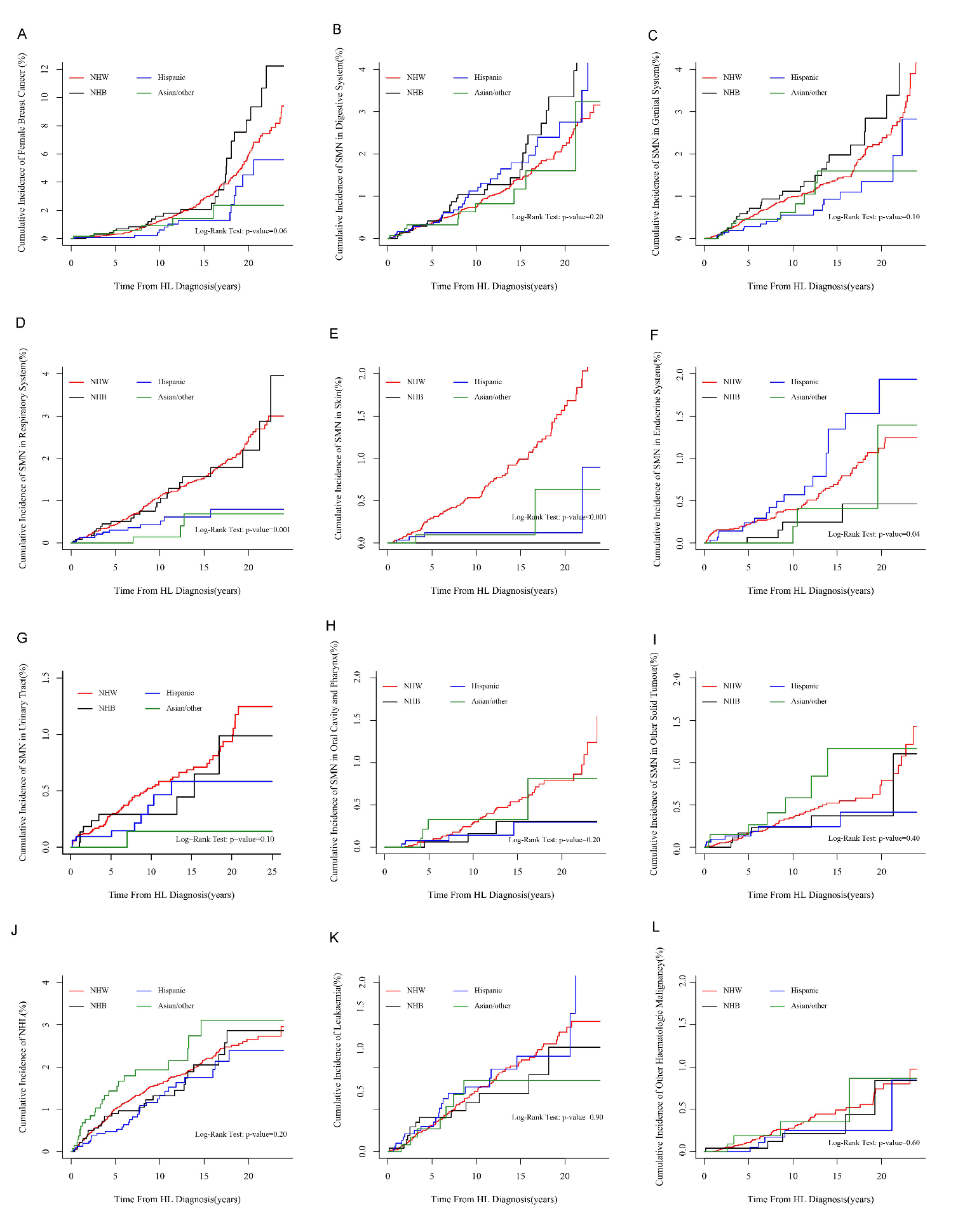

Supplement: Supplementary file 1 [file DataSheet_1.docx]
